# Association of Interleukin-18 Gene Polymorphism with Susceptibility to Visceral Leishmaniasis in Endemic Area of Bihar, an Indian Population

**DOI:** 10.1155/2014/852104

**Published:** 2014-10-22

**Authors:** Dinesh Kumar, Puja Tiwary, Jaya Chakravarty, Shyam Sundar

**Affiliations:** Infectious Disease Research Laboratory, Department of Medicine, Institute of Medical Sciences, Banaras Hindu University, Varanasi, Uttar Pradesh 221 005, India

## Abstract

Interleukin-18 (IL-18) is a cytokine that mediates Th1 response by inducing interferon-gamma (IFN-*γ*) production in T cells and natural killer cells. Genetic polymorphisms in the IL-18 gene have been found to be associated with its expression in cancer, tuberculosis, HBV infection, and various other diseases. Lower plasma level of IL-18 in visceral leishmaniasis (VL) patients might be associated with polymorphisms in the regulating or coding region of the gene. Three single nucleotide polymorphisms (SNPs), rs1946519 (−656 G/T) and rs187238 (−137 G/C) in the promoter region and rs549908 (+105 A/C) in the codon region, were genotyped in 204 parasitological confirmed VL patients and 267 controls with no past history of VL. For each locus, polymerase chain reaction (PCR) followed by restriction digestion was performed. IL-18 expression in peripheral blood mononuclear cells (PBMC) collected from VL patients and controls was measured by quantitative real-time RT-PCR. Distribution of G allele at position −656 (*P* < 0.0001) and double haplotypes GGC/GGA (*P* = 0.05) were found to be significantly associated with controls while genotypes TT (*P* < 0.0001) and single haplotypes TGA (*P* = 0.0002), with cases. The inheritance of G allele at the position −656 might be considered as a protective allele for VL.

## 1. Introduction

Visceral leishmaniasis (VL) is a potentially fatal infectious disease caused by the intracellular protozoan parasites* Leishmania donovani* and* Leishmania infantum *(*chagasi*). VL is responsible for significant morbidity and mortality in the Indian subcontinent and Brazil. The secondary infections, responsible for many deaths may be due to the immunocompromised status of VL patients. The mechanisms preventing clearance of the infection and underlying the predisposition of VL patients to secondary infections are not well known.

A key immunological feature of VL is the inability of peripheral blood mononuclear cells (PBMCs) to proliferate or to produce interferon-*γ* (IFN-*γ*) in response to leishmanial antigens [1, 2]. Interleukin-18 (IL-18) is a member of the IL-1 cytokine family and plays an important role in both kinds of immunity, innate and acquired. Initially it was characterized by its capacity to promote Th1 responses in synergy with IL-12 [3]. Th1-type immunity triggers enhanced leishmanicidal activity in infected macrophages, thus playing a protective role. IL-18 has been described for broader properties in the acquired immune responses. It was shown to induce proliferation of T-cell, IL-12R*α* expression, IFN-*γ*, TNF-*α*, and GM-CSF production by Th1 clones [4]. However, at early stages of T cell differentiation, IL-18 can promote either Th1 or Th2 responses independently of IL-4 or IL-12, suggesting its broader role in functional T cell differentiation [5].

IL-18 also was shown to induce IFN-*γ* production by NK cells, suggesting its involvement in the innate responses [6]. After exposure to IFN-*γ*, intracellular killing in macrophages involves mono-oxidative mechanisms through the generation of nitric oxide and its metabolites from L-arginine in presence of nitric oxide synthase [7]. Previous studies demonstrated that IL18 upregulates the expression of intracellular adhesion molecule-1 (ICAM-1) and vascular cell adhesion molecule-1 (VCAM-1) on the endothelial cells that play important role in inflammation process [8].

Several studies in other disease models have shown the association of differential plasma level of IL-18 with the single nucleotide polymorphisms (SNPs) in its encoding region [9–11]. Such studies clearly point towards possibility of the gene polymorphisms in affecting susceptibility or protection towards the same disease in different individuals [12–14]. Recently, Moravej et al. has found the association of IL-18 gene polymorphism with VL in Iranian population [15]. The importance of IL-18 in the defence against VL and the effect of gene polymorphism on its production in other diseases inspired us to investigate the probable association between IL-18 gene polymorphism and VL disease susceptibility in Indian population.

## 2. Materials and Methods

### 2.1. Subjects

A total of 471 subjects were enrolled for the study. 204 parasitologically confirmed VL patients were admitted at Kala-Azar Medical Research Centre in Muzaffarpur, Bihar. Characteristics of all the subjects are shown in Table 1. The inclusion criteria included subjects aged more than two years with sign and symptoms of VL and confirmed by microscopic identification of parasite in their splenic biopsies. Healthy controls were the subjects living in the endemic area with no history of VL, aged more than 18 years and also with negative serology.

The study was approved by the Ethical Committee of the Institute of Medical Sciences, Banaras Hindu University, Varanasi. Written informed consents were obtained from all participating subjects.

### 2.2. Sample Collection and Storage

Peripheral blood was drawn from each subject and collected in citrate vacutainers (BD Biosciences) and stored at 4°C until DNA isolation. 5 mL blood was drawn from 13 cases and 13 controls for isolation of PBMC and stored in RNAlater (Qiagen, Hilden) at −80°C until RNA extraction.

### 2.3. DNA and RNA Extraction

DNA was isolated from 200 *μ*L peripheral blood using QIAamp Blood DNA mini kit (Qiagen) while RNA was isolated from PBMC using RNeasy mini kit (Qiagen, Hilden) as per the manufacturer's protocol. All the samples were quantified using NanoDrop 2000c and aliquoted in triplicate to avoid damage during repeated freeze thawing and stored.

### 2.4. IL-18 Expression Study

1 *μ*g RNA was isolated from PBMC, was reverse transcribed in a 20 *μ*L reaction using High Capacity cDNA Reverse Transcription Kit (Invitrogen USA), and was diluted 10 times with RNase free water for final concentration of 5 ng/*μ*L. TaqMan gene expression assays (Applied Biosystems USA) for IL-18 (Hs01038788) were used to perform expression studies with 18S rRNA (P/N 4319413E) used as an endogenous control and run on 7500 Real-Time PCR System (Applied Biosystems USA). All samples were run in duplicate along with no template master mix as negative controls in each plate. Results were analysed by 7500 software v.2.0.1 (Applied Biosystems) and Graph pad prism 5. Unpaired two-tailed *t*-tests were used to compare IL-18 expression level in cases and controls.

### 2.5. Genotyping

PCR-RFLP based method was used to detect polymorphism in IL-18 gene. Three SNPs, rs1946519 (−656 G/T) and rs187238 (−137 G/C) of promoter region and rs549908 (+105 A/C) of codon region, were selected for the study. For each polymorphism, a specific PCR-RFLP was done using method described previously [13] with few modifications (Table 2). PCR was performed in 25 *μ*L reaction mixture using 100 ng genomic DNA with 1.5 U Taq DNA polymerase (New England Bio labs, UK) supplemented with 10X Taq DNA polymerase buffer, 1.5 mM MgCl_2_, 25 *μ*M dNTPs, and 10 pM of forward and reverse primers. Each amplified product was digested by specific restriction enzymes (Fermentas) and electrophoresed on a 3% (w/v) ethidium bromide (Merck, Mumbai) stained agarose gel (Sigma-Aldrich, St. Louis, USA) and visualised on UV transilluminator.

### 2.6. Statistical Analysis

Allele and genotype frequencies of three SNPs were estimated by observing the digested fragments. All the data was entered into EPI info 2,000. *χ* ^2^ test, odds ratio, and 95% confidence interval were calculated by GraphPad prism 5 and SPSS software version 16. SNPStats software was used to estimate the haplotypes frequencies and Hardy-Weinberg equilibrium. The *P* values < 0.0015 were considered as statistically significant after Bonferroni correction for multiple tests. The linkage disequilibrium (LD) measurings, *R* ^2^ and *D*′, were estimated by LD2SNPing program V 2.0 (http://bio.kuas.edu.tw/LD2SNPing/).

## 3. Results and Discussion

**Figure 1:**
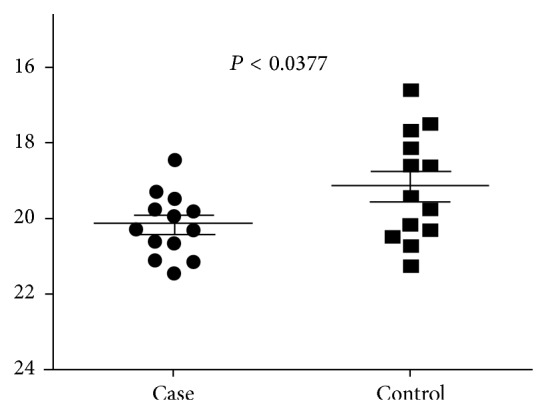
The expression level of IL-18 gene is determined by RT-PCR in cases of VL and healthy controls. The solid line represents cutoff value and dot represents individual subjects.

Quantitative real-time RT-PCR was done to observe expression level of IL-18 in PBMC collected from 13 patients and 13 controls and it was found significantly upregulated in controls (*P* < 0.0377) (Figure 1).

**Figure 2:**
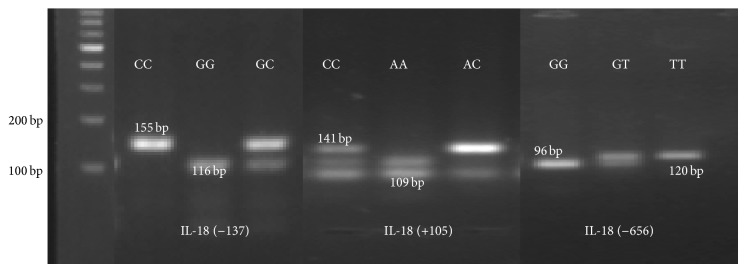
The agarose gel electrophoretic patterns of IL-18 gene polymorphism.

PCR-RFLP methods were used to analyse the IL-18 gene SNPs and the electrophoretic pattern was shown in Figure 2.

The frequency of G allele at position −656 was found significantly higher in the control group as compared to cases (70.60 versus 56.86%, *P* < 0.0001, OR = 0.549, 95% CI = 0.419–0.719 and study power 99.2%), while frequency of G allele at −137 was found significantly higher in cases (78.19 versus 71.91% *P* = 0.02, OR = 1.40, 95% CI = 1.03–1.89 and study power 59.42%). At position +105 both A and C alleles were equally distributed in cases and controls (76.03%, *P* = 0.80, OR = 1.03, 95% CI = 0.76–1.40 and study power 3.70%). The distributions of genotypes were according to the Hardy-Weinberg equilibrium.

At the position −656 the TT genotype frequency was significantly higher in cases while at other positions −137 and +105 no significant difference was found between cases and controls. The genotypes −656 GG, −137 GG, +105 AA were frequently distributed in both cases and controls (Table 3). The distribution of haplotypes in IL-18 polymorphism showed that the frequency of GGA at positions −656, −137, and +105 was higher in control (38.31%, *P* = 0.23, OR = 0.792, 95% CI = 0.54–1.161 and study power 22.12%). GGA haplotypes were the most frequently distributed in all the subjects, while haplotypes TGA were higher in cases (28.33 versus 14.49%, *P* = 0.0002, OR = 2.30, 95% CI = 1.47–3.68 and study power 95.52%).

32 double haplotypes were constructed in the present study. The frequencies of the commonly distributed haplogenotypes were shown in Table 4 and those haplotypes having frequencies less than 5% were omitted from the table. The distribution of TGA/GGA haplogenotypes was found to be higher in cases (18.63 versus 11.24%, *P* = 0.02, OR = 1.80, 95% CI = 1.07–3.03 and study power 61.58%), but the *P* value could not tolerate Bonferroni correction. The haplogenotypes GGC/GGA were found more frequently distributed in controls (11.16 versus 6.37%, *P* = 0.05, OR = 0.05, 95% CI  =  0.26–1.08 and study power 49.03%).

**Figure 3:**
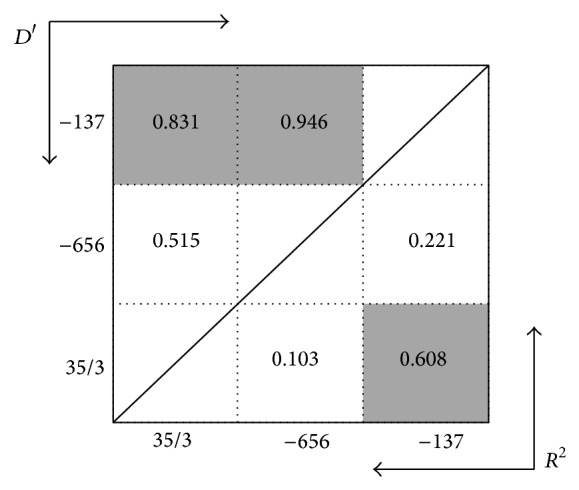
Linkage disequilibrium (LD) plot of IL-18 polymorphism in *R* ^2^ and D′ value in VL patients. Dark color cells represent high LD values.

**Figure 4:**
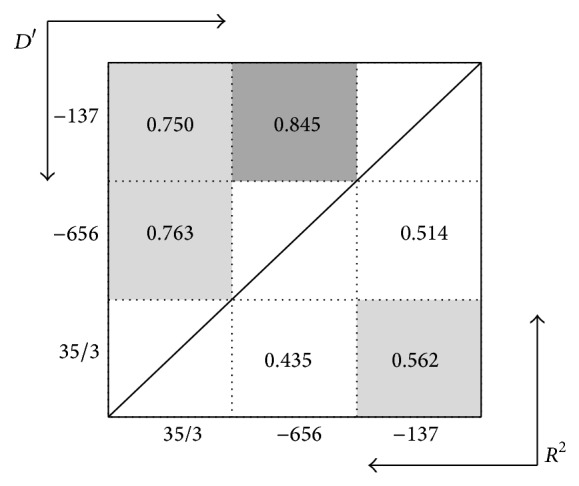
Linkage disequilibrium (LD) plot of IL-18 polymorphism in *R* ^2^ and D′ value in controls. Dark color cells represent high LD values.

The strong LDs were detected between all SNPs in both controls and cases (Figures 3 and 4).

IL-18 has been classified in the IL-1 family in virtue of structural similarity to IL-1*β*. Its ability to induce IFN-*γ* from Th1 and NK cell separates it from IL-1. Thus IL-18 was originally identified as an IFN-*γ* inducing factors. Some studies revealed that IL-18 induces the production of Th2 cytokines from T-cell, NK cell, basophiles, and mast cells [16–20]. Moreover, IL-18 can directly enhance the proliferation and cytotoxicity of Tc cell and NK cell [21–24]. Thus IL-18 is a pleotropic cytokine which regulates both natural and acquired immune response. The major source is macrophages, although many other are also capable of producing IL-18 [25, 26].

Although the production of IL-18 is affected by many factors, it could also be affected by genetic variants in its promoter region. It is tempting to speculate that polymorphisms in the IL-18 promoter could affect its expression. Changes in IL-18 expression levels might disturb the balance between Th1 and Th2 cytokine responses.

Considering the involvement of IL-18 in affecting the course of infection, investigating the common genetic variants in IL-18 gene could provide important genetic determinants of VL. This can lead to new immune-based therapies by targeting cytokine signalling pathways. Taking the role of IL-18 in VL pathogenesis and the effect of known polymorphisms in the promoter region in affecting its expression levels, we wanted to investigate the relationship between IL-18 gene variants and susceptibility and/or protection to* Leishmania *infection in North Indian population.

Previously studies revealed that the higher production of IL-18 and IFN-*γ* is associated with the presence of nucleotides G and A at the positions −137 and +105, respectively [9–11]. So we assumed that these alleles must be more frequent in control group as compared to VL cases, but we did not get any significant differences in the distribution of these alleles between cases and controls. Contrary to the two aforementioned polymorphic sites, the frequency of G allele at the position −656 was significantly higher in controls than the cases. Thus, G allele at −656 could be considered as a protective factor for the VL in our population.

In addition to the genotypes and alleles, inherited combination of SNPs and polymorphic haplotypes can influence predisposition to different diseases. The frequency of haplotypes GGA was higher in controls, while frequency of TGA was significantly higher in cases of VL. The inheritance of GGC/GGA haplotypes was significantly distributed in control (*P* < 0.052), while TGA/GGA was more frequent in cases (*P* < 0.02). It can be supposed that the inheritance of TGA/GGA haplogenotypes might be major genetic risk factor for the occurrence of VL.

Previously, other published reports revealed association of IL-18 gene polymorphisms with susceptibility to VL in Iranian population, with different allele [15]. Because of population specific differences in allele and disease frequency, the genetic associations become more meaningful when replicated in varied ethnic populations. Likewise, several studies have reported the association of IL-18 gene variants and susceptibility to infectious and noninfectious diseases like* Brucella* infection [27], HBV infection [28], type-1 diabetes [29], periodontitis [13].

## 4. Conclusion

The frequency of G allele at position −656 (rs1946519) was significantly associated with protection to VL in our north eastern population. Thus presence of G allele at position −656 can be considered as a potential candidate as protective allele for VL.

## Figures and Tables

**Table 1:** Characteristics of the subjects.

Characteristic	Case (*n* = 204)	Control (*n* = 267)
Age (mean ± SD) (yr)	24.9 ± 12.1	35.2 ± 12.6
Sex (no. males/no. females)	94/110	124/143
Fever history (mean ± SD) (days)	45.5 ± 47.7	
Spleen size (mean ± SD) (cm)	3.80 ± 1.92	
HIV status	Negative	Negative

**Table 2:** Polymerase chain reaction (PCR) primers and condition for IL-18 gene amplification.

SNPs	PCR primers	Annealing temperature (°C)	Restriction enzymes	Fragment sizes (bp)
+105 (A/C)	F: AGATTTAATGTTTATTGTAGAAAACCTGGGACTC R: CAGTCATATCTTCAAATAGAGGCCG	55	*Dde I *	A: 109 + 32 C: 141

−656 (G/T)	F: AGGTCAGTCTTTGCTATCATTCCAGG R: CTGCAACAGAAAGTAAGCTTGCGGAGAGG	60	*Mwo I *	G: 96 + 24 T: 120

−137 (G/C)	F: CACAGAGCCCCAACTTTTTACGGCAGAGAA R: GACTGCTGTCGGCACTCCTTGG	60	*Mbo II *	G: 116 + 39 C: 155

**Table 3:** IL-18 genotype and alleles frequencies in VL cases and controls.

Genotypes and alleles	Patient	*n* (%)	Control	*n* (%)	*χ* ^2^	*P* value	OR (95% CI)	Study power
**+105**								
Genotypes								
AA	114	55.88	145	54.31	0.116	0.7335	1.066 (0.739–1.538)	4.82%
CC	5	2.45	6	2.25	0.021	0.8846	1.093 (0.329–3.633)	2.68%
AC	85	41.67	116	43.45	0.149	0.6989	0.930 (0.643–1.345)	5.42%
Alleles								
A	313	76.72	406	76.03	0.060	0.8062	1.039 (0.767–1.407)	3.70%
C	95	23.28	128	23.97				

−**656**								
Genotypes								
GG	89	43.63	133	49.81	1.776	0.1827	0.780 (0.541–1.125)	26.42%
TT	61	29.90	23	8.61	35.76	<0.0001	4.525 (2.685–7.628)	100%
GT	54	26.47	111	41.57	11.59	<0.0007	0.506 (0.341–0.751)	93.11%
Alleles								
G	232	56.86	377	70.60	19.1	<0.0001	0.549 (0.419–0.719)	99.20%
T	176	43.14	157	29.40				

−**137**								
Genotypes								
GG	128	62.75	136	50.94	6.546	0.0105	1.622 (1.119–2.353)	72.77%
CC	13	6.37	19	7.12	0.101	0.7507	0.888 (0.428–1.844)	4.46%
GC	63	30.88	112	41.95	6.064	0.0138	0.618 (0.421–0.908)	69.55%
Alleles								
G	319	78.19	384	71.91	4.812	0.0283	1.40 (1.036–1.893)	59.42%
C	89	21.81	150	28.09				

**Table 4:** Frequently occurrence of IL-18 single and double haplotypes distributions in cases and controls.

Haplotypes	Cases	Control	*χ* ^2^	*P* value	OR (95% CI)	Study power
Single haplotypes						
GGA	67 (32.97)	102 (38.31)	1.444	0.2296	0.792 (0.54–1.161)	22.12%
TGA	58 (28.33)	39 (14.49)	13.52	0.0002	2.333 (1.477–3.684)	95.52%
GCA	23 (11.06)	36 (13.67)	0.5148	0.4731	0.785 (0.449–1.375)	12.96%
GGC	19 (9.45)	40 (14.94)	3.39	0.0656	0.594 (0.334–1.058)	42.71%
TCA	9 (4.35)	26 (9.56)	4.769	0.029	0.430 (0.196–0.945)	58.07%
TGC	15 (7.43)	11 (4.17)	2.318	0.1279	1.845 (0.832–4.089)	33.77%
GCC	7 (3.38)	10 (3.68)	0.03276	0.8564	0.916 (0.34–2.467)	2.81%
TCC	6 (3.02)	3 (1.18)	2.038	0.1534	2.608 (0.663–10.263)	30.53%
Double haplotypes						
GGA/GGA	22 (10.78)	39 (14.61)	1.49	0.22	0.70 (0.40–1.23)	22.75%
TGA/GGA	38 (18.63)	30 (11.24)	5.11	0.02	1.80 (1.07–3.03)	61.58%
GCA/GGA	15 (7.35)	28 (10.49)	1.36	0.242	0.67 (0.35–1.30)	21.02%
GGC/GGA	13 (6.37)	31 (11.61)	3.74	0.052	0.05 (0.26–1.08)	49.03%

## References

[1] Sacks D. L., Lal S. L., Shrivastava S. N., Blackwell J., Neva F. A. (1987). An analysis of T cell responsiveness in Indian Kala-azar. *The Journal of Immunology*.

[2] White A. C., Castes M., Garcia L., Trujillo D., Zambrano L. (1992). *Leishmania chagasi* antigens recognized in cured visceral leishmaniasis and asymptomatic infection. *American Journal of Tropical Medicine and Hygiene*.

[3] Munder M., Mallo M., Eichmann K., Modolell M. (1998). Murine macrophages secrete interferon *γ* upon combined stimulation with interleukin (IL)-12 and IL-18: a novel pathway of autocrine macrophage activation. *The Journal of Experimental Medicine*.

[4] Okamura H., Kashiwamura S.-I., Tsutsui H., Yoshimoto T., Nakanishi K. (1998). Regulation of interferon-*γ* production by IL-12 and IL-18. *Current Opinion in Immunology*.

[5] Xu D., Trajkovic V., Hunter D., Leung B. P., Schulz K., Gracie J. A., McInnes I. B., Liew F. Y. (2000). IL-18 induces the differentiation of Th1 or Th2 cells depending upon cytokine milieu and genetic background. *European Journal of Immunology*.

[6] Takeda K., Tsutsui H., Yoshimoto T., Adachi O., Yoshida N., Kishimoto T., Okamura H., Nakanishi K., Akira S. (1998). Defective NK cell activity and Th1 response in IL-18-deficient mice. *Immunity*.

[7] Green S. J., Meltzer M. S., Hibbs J. B., Nacy C. A. (1990). Activated macrophages destroy intracellular Leishmania major amastigotes by an L-arginine-dependent killing mechanism. *Journal of Immunology*.

[8] Morel J. C. M., Park C. C., Woods J. M., Koch A. E. (2001). A novel role for interleukin-18 in adhesion molecule induction through NF *κ*B and phosphatidylinositol (PI) 3-kinase-dependent signal transduction pathways. *Journal of Biological Chemistry*.

[9] Liang X. H., Cheung W., Heng C. K., Wang D. Y. (2005). Reduced transcriptional activity in individuals with IL-18 gene variants detected from functional but not association study. *Biochemical and Biophysical Research Communications*.

[10] Giedraitis V., He B., Huang W.-X., Hillert J. (2001). Cloning and mutation analysis of the human IL-18 promoter: a possible role of polymorphisms in expression regulation. *Journal of Neuroimmunology*.

[11] Arimitsu J., Hirano T., Higa S., Kawai M., Naka T., Ogata A., Shima Y., Fujimoto M., Yamadori T., Hagiwara K., Ohgawara T., Kuwabara Y., Kawase I., Tanaka T. (2006). IL-18 gene polymorphisms affect IL-18 production capability by monocytes. *Biochemical and Biophysical Research Communications*.

[12] Kalani M., Rasouli M., Moravej A., Kiany S., Rahimi H. R. (2011). Association of interleukin-15 single nucleotide polymorphisms with resistance to brucellosis among Iranian patients. *Tissue Antigens*.

[13] Folwaczny M., Glas J., Török H.-P., Tonenchi L., Paschos E., Bauer B., Limbersky O., Folwaczny C. (2005). Polymorphisms of the interleukin-18 gene in periodontitis patients. *Journal of Clinical Periodontology*.

[14] Moravej A., Rasouli M., Kalani M., Asaei S., Kiany S., Najafipour S., Koohpayeh A., Abdollahi A. (2012). IL-1*β* (-511T/C) gene polymorphism not IL-1*β* (+3953T/C) and LT-*α* (+252A/G) gene variants confers susceptibility to visceral leishmaniasis. *Molecular Biology Reports*.

[15] Moravej A., Rasouli M., Asaei S., Kalani M., Mansoori Y. (2013). Association of interleukin-18 gene variants with susceptibility to visceral leishmaniasis in Iranian population. *Molecular Biology Reports*.

[16] Yoshimoto T., Tsutsui H., Tominaga K., Hoshino K., Okamura H., Akira S., Paul W. E., Nakanishi K. (1999). IL-18, although antiallergic when administered with IL-12, stimulates IL-4 and histamine release by basophils. *Proceedings of the National Academy of Sciences of the United States of America*.

[17] Yoshimoto T., Mizutani H., Tsutsui H., Noben-Trauth N., Yamanaka K.-I., Tanaka M., Izumi S., Okamura H., Paul W. E., Nakanishi K. (2000). IL-I 8 induction of IgE: dependence on CD4^+^ T cells, IL-4 and STAT6. *Nature Immunology*.

[18] Hoshino T., Wiltrout R. H., Young H. A. (1999). IL-18 is a potent coinducer of IL-13 in NK and T cells: a new potential role for IL-18 in modulating the immune response. *The Journal of Immunology*.

[19] Nakanishi K., Yoshimoto T., Tsutsui H., Okamura H. (2001). Interleukin-18 is a unique cytokine that stimulates both Th1 and Th2 responses depending on its cytokine milieu. *Cytokine and Growth Factor Reviews*.

[20] Hoshino T., Yagita H., Ortaldo J. R., Wiltrout R. H., Young H. A. (2000). *In vivo* administration of IL-18 can induce IgE production through Th2 cytokine induction and up-regulation of CD40 ligand (CD154) expression on CD4^+^ T cells. *European Journal of Immunology*.

[21] Netea M. G., Kullberg B. J., Verschueren I., van der Meer J. W. M. (2000). Interleukin-18 induces production of proinflammatory cytokines in mice: no intermediate role for the cytokines of the tumor necrosis factor family and interleukin-1 beta. *European Journal of Immunology*.

[22] Dinarello C. A., Fantuzzi G. (2003). Interleukin-18 and host defense against infection. *Journal of Infectious Diseases*.

[23] Helmby H., Grencis R. K. (2002). IL-18 regulates intestinal mastocytosis and Th2 cytokine production independently of IFN-*γ* during *Trichinella spiralis* infection. *The Journal of Immunology*.

[24] Wei X.-Q., Leung B. P., Arthur H. M. L., McInnes I. B., Liew F. Y. (2001). Reduced incidence and severity of collagen-induced arthritis in mice lacking IL-18. *The Journal of Immunology*.

[25] Akita K., Ohtsuki T., Nukada Y., Tanimoto T., Namba M., Okura T., Takakura-Yamamoto R., Torigoe K., Gu Y., Su M. S.-S., Fujii M., Satoh-Itoh M., Yamamoto K., Kohno K., Ikeda M., Kurimoto M. (1997). Involvement of caspase-1 and caspase-3 in the production and processing of mature human interleukin 18 in monocytic THP.1 cells. *Journal of Biological Chemistry*.

[26] Okamura H., Tsutsui H., Komatsu T., Yutsudo M., Hakura A., Tanimoto T., Torigoe K., Okura T., Nukada Y., Hattori K., Akita K., Namba M., Tanabe F., Konishi K., Fukuda S., Kurimoto M. (1995). Cloning of a new cytokine that induces IFN-*γ* production by T cells. *Nature*.

[27] Rasouli M., Kalani M., Moravej A., Kiany S. (2011). Interleukin-18 single nucleotide polymorphisms contribute to the susceptibility to brucellosis in Iranian patients. *Cytokine*.

[28] Zhang P.-A., Wu J.-M., Li Y., Yang X.-S. (2005). Association of polymorphisms of interleukin-18 gene promoter region with chronic hepatitis B in Chinese Han population. *World Journal of Gastroenterology*.

[29] Tavares N. A. C., Santos M. M. S., Moura R., Araújo J., Guimarães R., Crovella S., Brandão L. (2013). Interleukin 18 (IL18) gene promoter polymorphisms are associated with type 1 diabetes mellitus in Brazilian patients. *Cytokine*.

